# "The Hospital" Nursing Mirror

**Published:** 1900-08-25

**Authors:** 


### "The Hospital" Nursing Mirror.

Being the Nursing Section of "The Hospital."

[Contributions for this Section of "The Hospital" should be addressed to the Editor, The Hospital, 28 & 29, Southampton Street, Strand, London, W.C., and should have the word "Nursing" plainly written in left-hand top corner of the envelope.]

### Notes on News from the Nursing World.

### NURSING IN MILITARY HOSPITALS.

We direct attention to two very interesting articles in another part of the paper—one on the nursing arrangements at Netley, and the other in reference to nursing by orderlies. The sister who has been interviewed by a correspondent on the latter question makes a very serious charge against the orderlies. She says that it is "by no means an unusual occurrence" to find an orderly asleep on duty; and "occasionally" the orderlies "are more or less under the influence of drink." She adds that the excessively long hours explain sleeping on duty. But the hours should not be excessively long; and as to the graver allegation an orderly who gets drunk on duty should be dismissed, instead of "reinstated in the same ward" when he has been locked up for a day or two. A very fair view is taken of the nursing arrangements at Netley. The quarters of the sisters are certainly capable of improvement, and the system of allowances described will doubtless be altered when the scheme of reorganisation, foreshadowed in The Hospital on August 11th, is enforced.

### MORE WAR NURSES.

The dispatch of nurses to the seat of the war continues, though the latest intimation from the Secretary of State for War suggests that the authorities have arrived at the conclusion that the supply is no longer inadequate. We are officially informed that Sisters A. B. Jackson and J. L. Carlyon, of the Army Nursing Service Reserve, embarked for South Africa on Saturday last. Sister A. R. Chitty, whose departure we announced last week, was trained at the Alfred Hospital, Melbourne, and then took the post of staff nurse at the General Hospital, Birmingham. After a year's service she was promoted to the sistership of a male accident ward, which she held for 18 months, resigning in order to do duty for three months in the operating theatre. She left Birmingham to be sister of the male medical ward at the Taunton and Somerset Hospital, and Taunton in order to take up the appointment of nurse-matron of the London Throat Hospital, Great Portland Street, a position she retained for two years and a quarter. Miss Chitty was subsequently, for nearly a year, matron of the General Hospital, Stroud.

### THE SISTERS OF MERCY AT MAFEKING.

The public will not turn a deaf ear to the appeal which has been issued by Lady North, from Wroxton Abbey, Banbury, on behalf of the Sisters of Mercy at Mafeking, whose convent, chapel, and garden were wrecked during the bombardment. The nature of the work done by these ladies during the siege is attested by General Baden-Powell, who, in a letter to the Reverend Mother Teresa, enclosing £98 15s., the proceedings of a raffle, says: "The mere money does not in any way represent adequately the lasting gratitude of the community towards yourself and the Sisters for the valuable, self-devoting services you have rendered to the sick and wounded in hospital, nor our sympathy for your losses and trouble, incident to the siege." We are confident that the losses incurred by these devoted women, who spared no effort to supply as far as possible the place of nurses during the bombardment of Mafeking, will be covered by the contributions of the grateful.

### A VOLUNTEER NURSE AT MAFEKING.

The matron at Guy's Hospital writes in reference to a paragraph under the heading of "A Volunteer Nurse at Mafeking" in our issue of August 11th, to point out that the paying probationers who are received at Guy's Hospital, enter for twelve, not for three, months, as the paragraph for August 11th seemed to suggest.

### TYPHOID FEVER AND ICE.

At a time when the nursing of typhoid in South Africa is exciting so much interest the experience of a Red Cross nurse during the Spanish-American war of 1898 may be interesting. She describes a day in a typhoid ward as follows:—

"After a fitful and unfreshing sleep, snatched after prolonged battling with mosquitos, we were awakened by the curious street cries of an old Southern city, tattered negro boys chanting their wares with a musical cadence strange to northern ears. We rose and bathed hastily, and, while putting on clean clothing, reflected sadly that by ten a.m. we should be dishevelled-looking creatures, dripping with perspiration. The night nurses, weary and hollow-eyed, were glad to be relieved. The ward looked exactly the same as when we left it the previous night. The man in the first bed was still muttering in delirium, with a fixed glare in his eyes. The poor sun-struck patient in the opposite bed was still weeping over the loss of a five-dollar bill stolen from under his pillow. The greeting of the convalescents was loud and cheerful, for the arrival of the day nurses meant 'breakfast,' and to a recovering 'typhoid' meals are the only consideration. After breakfast was over we took temperatures, and found that we should be busy with ice baths till noon. As portable bath tubs are unknown in these regions, we spread a rubber sheet on the bed, packed the patient with ice, covered him with blanket, and sponged vigorously for half-an-hour. Oh, how the poor fellows did hate it! In the afternoon, after things were fairly quiet, we wrote letters from dictation from the soldiers to their anxious friends. At four the ice bathing began again. Towards supper time the convalescents' eyes turned anxiously to the clock; the coloured ward-maid at length appeared, with bare feet, bearing an immense wooden tray on her head. She was greeted with great acclaim, the most popular person in the community for the moment. The nurses were sometimes thought slow and stupid, apt to delay in serving, and with limited ideas of quantity. Dividing the ice-cream was a very delicate operation, watched eagerly by hundreds of eyes. At last seven o'clock struck, and then a lot of fagged and rather dirty-looking nurses crawled home through the dusty streets to their supper."

### GUARDIANS AND THE LOCAL GOVERNMENT BOARD.

Mr. Dawson, the Local Government Board Inspector for the district, has expressed his regret that the Tynemouth Board of Guardians have not increased the nursing staff at the workhouse. He himself, at the suggestion of the Local Government Board, urged last year that this should be done; but the Guardians ignored the suggestion, and allowed the number of patients to each nurse to rise as high as 30. We do not, of course, assert that the Local Government Board are infallible, or that the dictum of the inspectors ought always to be accepted. But there is no doubt that, as a rule, the officials of the Board do not propose additions to the nursing staff of a workhouse unless they are obviously required.

### A PICNIC FOR FEVER NURSES.

Exception has been taken by a member of the Bradford Corporation to an item of £27 11s., the expense incurred in providing a picnic for the nurses at the Fever Hospital. The member described it as "a rather dangerous precedent." But the answer of the Health Committee, who sanctioned the outlay, was that the case was not without precedent, and that the picnic was given at the cost of the Corporation in consideration of the arduous labours of the nurses during a severe fever epidemic. It is easy to make too free with the money of the ratepayers, but in such circumstances as these we do not think that the Bradford Corporation can be blamed. It is manifestly to the interest of the ratepayers that the nurses at the Fever Hospital should not break down in health, and the little outing they enjoyed may have been just the change they needed to keep them going.

### THE STOCKPORT GUARDIANS AND THEIR NURSES.

The frequent resignation of nurses has lately been a cause of trouble to the Stockport Board of Guardians, and in order to ascertain if the salaries question had anything to do with the matter, a return of salaries paid in neighbouring unions was moved for and obtained. From this return, it has been ascertained that in comparison with Chorlton, Ashton-under-Lyne, and Oldham, the officials of the Stockport Workhouse are well, and even excessively, paid. But, as Canon Maloney suggested at the meeting of Guardians which was held to consider the subject, it does not follow that these three workhouses should be taken as models. If the Stockport Guardians wish to keep their nurses they must make it worth their while to stay; and it is possible that more consideration for their comfort and convenience may be at least as much needed as sufficient salaries.

### A DISTRICT NURSE FOR RICHMOND, YORKSHIRE.

It has been decided to appoint a district nurse in connection with the new Victoria Nursing Institution at Richmond, Yorkshire. Miss Boultbee, who at a public meeting reviewed at length the probable working of the home which had been opened in Frenchgate, and which has already rendered good service, estimated that about 5,000 visits would require to be made in the year, and that the working of the institution would cost about £100. A representative committee was subsequently selected, the urgent necessity for the movement being illustrated by a member of the Corporation, who mentioned instances of people in Richmond "dying from want of proper nourishment and treatment." This, it may be hoped, will not occur when a nurse has been installed to provide for the wants of the sick poor.

### BELPER ISOLATION HOSPITAL.

A very reasonable proposal for dealing with the difficulties which have arisen in connection with Belper Isolation Hospital has been rejected by the Council. It was to the effect that the whole matter should be placed in the hands of the Local Government Board, and that they should be asked to send down an inspector to sift the charges which had been made, the idea being that an independent, impartial, and fearless authority would, in the words of one of the council, "lay bare the sore that has festered in the hospital since it has opened." As, according to the same speaker, "the whole of the committee are either prejudiced in favour of Miss Martin, the matron, or bitterly opposed to her," it looks as if nothing short of an independent inquiry could be satisfactory. The conflicting nature of the views held by the committee is shown by the fact that while one member said that the complaints were "all side issues and did not relate to the treatment of patients or nursing," another alleged that "they were made on points of nursing." Certainly, the policy of the committee in holding their sittings with closed doors has not been vindicated by the result.

### A LETTER IN SEASON.

At a recent meeting of the Holywell Board of Guardians a letter was read from Lady Mostyn, of Talacre, expressing her regret that the Guardians had declined to subscribe to the Gwespyr Villagers' Nursing Club. In the course of her communication she pointed out that the members of the club are working men, who subscribe a penny each weekly, and she also enclosed a long list of Welsh Unions which contribute to the maintenance of nurses in rural districts. Apparently, this timely remonstrance has had its effect. The Holywell Guardians have decided to ascertain the amount of the subscriptions given by other Boards; and if they possess the legal power to render the assistance suggested, it may be presumed that they will not withhold a measure of support to the Nursing Club, which is doing an excellent work.

### MRS. MALAPROP IN THE HOSPITAL.

A well-meaning visitor, inspecting the children's ward of a Midland hospital on a day which also happened to be the visiting day for the patient's friends, went over to a cot where a mother was anxiously bending over her child, a mite of two, with a broken leg. "Well," said the visitor, "the poor little thing looks very comfortable, but I shouldn't like a child of mine to come here." The sister-in-charge, who was justly proud of her pretty ward, and the appearance of her little patients, felt more than a trifle hurt; and the visitor, seeing she had made a mistake, endeavoured to correct it. "Not," she added, "but that I am *sure* they are much better looked after than they would be *at home*." The glare in the eye of the child's mother, when she heard that remark, was something to be remembered.

### SHORT ITEMS.

Miss Jessie (Trilby) Howard-Smith, parish nurse at All Saints, Upper Norwood, was married on Saturday at All Saints, Fulham, to Mr. P. Chester, of Upper Norwood. The bride was given away by Miss Heatley, matron of St. Clement's Nursing Home, Fulham, to which she was formerly attached.—Miss E. S. Owen, superintendent nurse of Stepney Union Infirmary, Bromley-by-Bow, writes that as she has not yet received any notice from the War Office that she has been chosen for service in South Africa, she cannot be the Miss Owen mentioned in our issue of last week.

### Lectures on Medicine to Nurses.

By H. A. Latimer, M.D. (Dunelm), M.R.C.S. Eng., L.S.A.Lond.; Consulting Surgeon, Swansea Hospital; Past President of the South Wales and Monmouthshire Branch of Brit. Med. Assoc.; Past President of the Swansea Medical Society; Hon. Life Member of the St. John Ambulance Association, &c.

### INCUBATION AND ONSET OF FEVERS—DEFINITION OF THE TERM "SIGN."

Whenever any poisonous material has entered the blood it naturally takes some time before it does such an injury to the body that the cry of the disturbed organs affected by it becomes such that we are aware of something going wrong within us. In the case of the specific fevers this period of latency of symptoms which intervenes between the lodgment of the infective agent in the body and the outbreak of general disturbance of the system, in response to the irritation it has produced, is known as the period of incubation: that is the technical word, but the term used by the public for the same thing is the "time of breeding." You will constantly be asked by people how long it takes for measles, scarlatina, or some other zymotic disease to breed, before definite symptoms inquired about show themselves. You can give a definite reply based on the tables of incubation periods, which appear in all books on medicine, but you will note that there is, in most cases, a considerable margin set down as to the time taken over the matter. Some of the diseases in question are fairly precise in declaring themselves within a statutory period, whilst others are very vague in this respect and may break out soon after exposure to infection, or may be postponed for quite a comparatively long time. Of the first order we may take scarlet fever, measles, and small-pox as examples. These are set down as having incubations of three to five or even eight days, of ten to fourteen days, and of twelve to fourteen days respectively, and my experience is that these times (with the exception of what I have said about scarlatina) are very accurately set forth for the majority of cases one meets with. Over and over again I have known the rash of measles show itself on the fourteenth day after infection. In by far the majority of cases of scarlet fever, according to my experience, the breeding time has been from two to four days, and I think it is very rarely postponed for a longer term. Small-pox keeps its engagement well, and has always the long suspense between infection and outbreak that I have noted above. But see how indefinite some of the others are in this matter: Chicken-pox, ten to sixteen days; diphtheria, two to ten days; rötheln, or German measles, seven to eighteen days, or even longer; mumps, ten to twenty-two days; enteric fever, seven to twenty-one days; typhus, five to fourteen days; whooping cough, seven to fourteen days. There is little wonder, then, that during the period of slightly disturbed health which often accompanies the incubation of fevers (not always—for some, as typhus, declare themselves with extreme abruptness) the medical attendant has to assume an expectant attitude and to exhibit a masterly inactivity. It is in this regard, I think, that modern medicine is so vastly superior to the old theory and practice. It waits for definite symptoms of disease, and husbands the patient's strength in all possible ways, recognising that diseases which run a definite course cannot be arrested by violent measures such as were resorted to when bleeding and the antiphlogistic diet were in fashion. As a matter of fact the incubative periods of disease are not marked by definite symptoms pointing to the exact nature of what will follow later on. At the most the disturbed blood, which is in process of being poisoned by bacteria, causes disturbances of function in the nervous system over the body, and this reveals itself by some varying degree of headache, loss of appetite, and general lassitude. When any ailment is present in an epidemic form one is on the look-out for cases in one's own practice, and it is possible to be very wise as to what is going to occur soon in a person of a suitable age who does not seem to be quite well; but when these diseases present themselves as sporadic cases it is not always possible to be quite wise until "after the event." Then it is possible to sympathise with the man who said he preferred to prophesy about anything *after* it had happened. What really excites my wonder when thinking of these matters is that any disease whatever should keep any kind of time in its incubative period. When I think of the varied degree of resistance to its inroads, due to susceptibility or insusceptibility, whether of hereditary or of a personal nature; to the greater or lesser degree of exposure to infection; and to the varying virulence of the infective agent itself, I am surprised that any kind of regularity exists in the matter.

After the period of incubation, about which I have been speaking, there ensues an onset or invasion of definite symptoms, and the disease itself makes its appearance. In those complaints where the characteristic rash is evolved soon after the rise of temperature which marks a fever, or with the outbreak of the complaint, this eruption—or exanthem, as it is called—is in itself of so definite a character that an experienced observer is able then and there to declare to the patient's friends the nature of the illness from which his patient is suffering. Such rashes are of so peculiar and defined a nature that when we meet with them we regard them as diagnostic, and term them "signs." Now a sign differs from a symptom in a very marked and peculiar manner. The latter is indefinite, and may be common to a variety of illnesses, whilst the former is definite and pronounced in character, and is significant of one disease and of one disease alone. Let me illustrate these points by some examples. A patient is suffering, for instance, from headache, loss of appetite, rise of temperature, as marked by the thermometer, and cough. All these symptoms are, as I have told you in a previous lecture, but the cries of suffering organs, and it requires but a small experience in the observation of invalids to tell the investigator that such symptoms are common to a variety of ailments from which people suffer. Not one of them is diagnostic in itself: not one of them points to a definite illness. To ascertain the real nature of the disease from which the patient is suffering the physician has to examine him thoroughly and to correlate the symptoms. He has, among other things, to carefully examine the chest, windpipe, and throat, to ascertain what part of the respiratory organs is affected, whereby cough has resulted. And it is only by a review of these points that he is enabled to arrive at a decision and to give a diagnosis. With another illustration you will see how a sign differs from a symptom. A child, let us say, is suffering from the symptoms detailed above—from headache, loss of appetite, rise of temperature, and from cough. Presently a rash makes its appearance upon the face, and then spreads over the body. The physician notes certain characters in this eruption; he notes that it is very marked on the forehead, at the roots of the hair, and behind the ears; that it consists of raised spots, which, by their union with others, form a crescent-shaped spot; and he says to himself, "Here is a sign." This rash is the peculiar product of measles. The general symptoms help him in forming his opinion as to what the patient is suffering from, but the rash is a sign which points to measles, and to measles alone. Most of the eruptions are "signs." Do not dismiss the subject from your mind as a nurse in one of my classes once did. After explanations similar to the ones I have just given you of the recurrings of symptoms and signs, I asked her, "What is a sign?" She glibly replied, "A red spot on the skin in typhoid fever." She had not applied her mind to the general question at issue, but had simply used the especial illustration which I had happened to have given her.

### The Nursing Arrangements of Military Hospitals.

### NETLEY.

By a Correspondent.

"Rather more than a fortnight ago," writes a correspondent, "Netley was startled and surprised by the threatened invasion of the hospital by fifty nursing sisters, who, it was stated, would replace fifty orderlies leaving immediately for the front. To the unofficial mind this was a most entertaining proposal, and amongst ourselves we apportioned the various duties that would under the circumstances fall to our share. There were candidates for 'coal fatigue,' major's and colonel's orderlies, stimulant orderly, corridor orderly, dinner-waggon orderly (one sister proposing to ride on the top as it was run along from end to end of the corridor). The question of bringing up the beer and lemonade and other and various stores involved serious consideration, and it was decided that help must be asked from the civil surgeon.

"But, as it turned out, our anxiety was needless; better counsels, fortunately, prevailed, and the War Office has decided on more workable innovations. It is rumoured, but only rumoured, that four untrained ladies are to come and act as probationers, working under the sisters; while, with the duties already mentioned, the orderlies undertake the cleaning of the wards, polishing floors, and everything in connection with the lavatories. Without structural alterations this last item could not be worked by women at Netley.

"It may sound strangely to civil nurses, but Tommy was in great and real distress at the prospect of being washed by sisters instead of orderlies, one poor fellow who was very helpless stoutly declaring that 'it wasn't work for a lady to do,' and asking most anxiously if the lady superintendent had said sister must wash him. Seeing how upset he was she wisely gave in. He did not, however, in the least object to other strictly nursing matters being done by the same sister, explaining that 'that was different, because he was ill.'

"The Present Staff.

"There are in time of peace 11 nursing sisters on the Netley staff; but the present pressure of work has increased the number to 17, the divisions being in many instances halved, giving a sister 45 to 50, instead of 90 to 100 patients to look after; and where cases have been heavy a second sister has, in addition, been put on to help. This applies to day duty.

"At night the divisions are generally managed by two sisters, one on the surgical and medical floors respectively. A 'corridor orderly' walks up and down and visits certain wards (specified by the doctor) opening off his corridor every half-hour, he being relieved every two hours. In wards where there are more serious cases special orderlies on two-hourly shifts are on duty all night.

"Each sister makes a round of every ward on her floor every two hours, and visits much more frequently those patients who are very ill.

"The 'Scullery,' and Corridor Walking.

"On day duty there are no fixed visiting times. When not actually performing her work, the sister sits in the 'scullery,' a room which is attached to every division in the hospital, where all drugs, dressings, poisons, stimulants, &c., are kept, and in which any sick cookery ordered by the doctor is done. This system of returning to the scullery involves a great amount of walking, some idea of which may be gathered from the fact that the corridors are from end to end a quarter of a mile long, and on night duty, a sister in doing her round once, must walk that distance in the corridor alone, without taking into consideration any entrance into the sixeeen wards under her charge. But if there is much walking there is a corresponding amount of fresh air, which amply compensates, and I think all are agreed that night duty at Netley is preferable to any in a civil hospital from the point of view of the nurse's comfort. There is, of course, the constant anxiety as to what may be happening in a ward at one end of the division while the sister is busy at the other. More orderlies would remedy this. The usual period for night duty is a month or less; sometimes it is extended to six or seven weeks.

"Day Duty.

"The hours on day duty are arranged rather differently to those of a civil hospital. In summer the sisters usually breakfast at 7·30 and go into their divisions at 8 a.m., but since the exodus of orderlies the day's work, rising, begins at 6.15 instead of 6.45. The return of the orderlies is eagerly anticipated. Dinner is served at 1.15 p.m., half-an-hour having been allowed for lunch in the morning. At 2 p.m. sisters return to their divisions till 2·45, when they are off duty till 5.45 every day, Sundays included, having left written reports for the 'afternoon Sister' as to the cases she is to visit during their three hours of absence. The work is always as restricted in quantity as possible, as the whole hospital is for the time under her sole charge. Day sisters come on duty again at 6.45, and remain till 8.30 p.m., leaving a full written report of instructions for the night sisters, who take over at 9 p.m., and leave again at 7.30 a.m. Sisters duties consist chiefly in taking temperature, giving medicines and stimulants, often a most formidable undertaking owing to the number of patients, doing of dressings, sick cookery on a small scale, and stocking of stores for ward use—though desires are very much curtailed by red tape—and finally by assisting with helpless cases. The work is by no means nominal; at any rate, in time of war. When the lady superintendent can so arrange, each sister has a half-day weekly off duty—from 11 or 12 in the forenoon till 8.30 p.m. Late passes are very rarely granted.

"Services for Soldiers.

"Opportunity of going to service is always given once on Sunday, and is taken out of duty hours. Tommy is provided with six services every Sunday, and two or three in the week. The special prayers for protection and help gain a wonderful amount in reality, where offered by soldiers who have fought and suffered for their country, and the National Anthem sung at the end of morning service every Sunday stirs one's blood, as, standing at attention, it rings out from those who, in the words of a new version of 'God Save the Queen,' have been strong to 'help and defend the right.'

"So far as any hospital can be pleasant, Netley should rank high in the good opinion of Tommy, for there is much freedom from restraint in many particulars; he may smoke n the wards and corridors to his heart's content; get up and go to bed when he pleases; sit and lie on the quilts! have his beer and lemonade bottles beside him, and drink when he sees fit—the quantity being fixed by the doctor. This freedom accounts for a great deal of the dirtiness and untidiness of the wards, which strikes every civilian nurse so forcibly at first; and demonstrates, what somehow one was not prepared for in the soldier, that Tommy, in common with all the male gender, is essentially an untidy person.

"The Sisters' Quarters.

"As regards the 'Sisters' Quarters,' there is very great room for improvement. The bedroom accommodation is exceedingly poor, consisting of five cubicles, six small and ill-furnished rooms, and two fair-sized rooms. The splendidly large and airy sitting-room, commanding a good view of Southampton Water and the New Forest, which is well supplied with easy chairs and sofas, suffers greatly from being also the dining-room, meals or their remains being always en

*evidence.* The system of allowances adds to the discomfort. Each sister provides and prepares her own tea between 4 and 5.30 p.m., and at breakfast also has to make her own tea. Butter, sugar, and tea for each individual is kept in the sitting-room, and the remarkable assortment of butter dishes, sugar basins, and teapots does not add to the picturesqueness of meals. Three shillings and sixpence weekly covers, or is supposed to cover, these and washing expenses for each sister. The military kit system introduced into domestic matters also strikes one as curious, *i.e.*, each sister is provided with a certain quantity of bed linen and towels, and is held responsible for them and their washing. In case of loss she is required to replace them. All this will give some idea of the contrast between civil and military hospital life. The latter is very popular with the majority, and the posts of Army Nursing Service seem to be in demand in spite of poor remuneration."

### NURSING BY ORDERLIES.

BY A CORRESPONDENT.

IN view of the changes contemplated at Netley the subject of "Nursing by Orderlies" is of great interest at the present moment. Talking with a Sister at a military hospital, I asked, "What do you think of 'nursing by orderlies?'"

"An orderly's duties embrace so much more than the care of the sick that "nursing" proper is reduced to a minimum where Tommy is concerned. An orderly is a porter, for he must carry coals, bring up dinners, fetch stores, &c. He is also a wardmaid, for he is required to see to the cleaning of the ward, scrubbing of lockers and floors. Then he is responsible for the whole equipment of the ward or tent, inclusive of bedding, crockery, and all other utensils used in it, and in case of loss or breakage must himself supply the deficiency. Nursing and cleaning fight for the orderly's attention, and too often both have little of it."

"Do you mean that the patients are neglected by the orderlies?"

"No, not deliberately, except in a few instances; but work is so arranged that the sick must suffer. Nursing is made subservient to military customs and routine. The patient is not the first consideration, and matters which react enormously on Tommy are apparently beneath consideration."

### SIX CHANGES IN TWELVE HOURS.

"To what do you refer?"

"First of all to the two-hourly shifts of duty. This takes place between six p.m. and six a.m. It is impossible that there can be real continuity in the nursing when during a period of 12 hours there are six changes in the orderlies. True, it is worked by three men coming on in rotation for two hours and being off for four; but this necessarily involves six changes. The three are most probably quite unequally proficient, one being totally untrained—a regimental man—one a first-class orderly, and the third just a beginner in the R.A.M.C. They are of varying temperaments, intelligence, and character. Sick Tommy must try to adapt himself to each if he is able. The orderly has possibly never seen his patients before, may not see them or the ward again for six weeks. He is there for four hours of one night spent in two shifts. He has no interest in his patients or his work. If he is conscientious he does his best under difficult conditions, if careless and indifferent his one idea is to get through his two hours with as little trouble to himself as possible. Two hours at a time, on guard or sentry, may be well enough, but it is surely not suitable in the sick room."

"But is not this perhaps arranged to meet the difficulties of a short-handed staff?"

"I do not think it can possibly be so. It is a most extravagant waste of labour materia . would surely work better to have one orderly in a ward from eight p.m. to six a.m. for six weeks at a time—he would know his cases, take an interest in his work, and generally perform his duties more intelligently and systematically, and Tommy would be more comfortable."

### ASLEEP ON DUTY.

"Have you in your experience found the orderlies attentive in their duties?"

After a moment's consideration the Sister answered, "I was prepared in a sense to find some lapses in conduct, but the by no means unusual occurrence of an orderly asleep on duty did come as a great shock; and occasionally they are more or less under the influence of drink. One cannot help contrasting the disgrace that would rightly attach to such behaviour in a nurse, and the easy way in which it is actually, though not on blue paper, condoned in an orderly. He may be locked up for a day or two, but he is reinstated in the same ward when released. The excessively long hours explain sleeping on duty, but with better management this could be avoided."

### TOMMY'S PREFERENCE.

"Do you think Tommy would prefer to be nursed only by sisters?"

"I am sorry to say I don't think so, at any rate not at first," replied the Sister.

"Why not?"

"Well, though it hurts me to say it, Private ——, a gentleman ranker, gave it as his opinion that 'Sisters were too patronising, and Tommy hated to be patronised.' Another point against female nursing in the Army is, and I speak from data, the soldiers' sense of delicacy. To whatever it be attributable, as patients they compare most favourably with civilians. Perhaps, too, the restraint of a lady's presence—beneficial as it might be—would explain a preference for orderlies which I believe exists; but on the other hand, Tommy most deeply appreciates all that is done for him by sisters."

"You mean, then, that the R.A.M.C. is a popular corps?"

"Certainly not, quite the contrary. I have never heard a good word from the soldiers in reference to it."

### A HIGHER STANDARD OF DUTY REQUIRED.

"What do you personally think of the orderlies?"

"There are good and bad, but most of them are indifferent. I think that men are naturally unfitted for sick nursing. Constant and conscientious attention to details essential to real nursing seems to be too much to expect from an orderly, I had almost said from any man. Greater care in selection of the men, and a higher standard of duty required from them by Army officials, would do much to make orderlies far more efficient. Things are allowed to be so slack, and dirtiness and slovenliness seem to be an integral part of an orderly's nature."

### Minor Appointments.

COVENTRY CITY HOSPITAL, FIRTH.—Miss Edith Firth has been appointed Charge Nurse. She was trained at Mill Road Infirmary, Liverpool, and has since been charge nurse at the Park Hospital, and charge nurse and night superintendent at the Northern Fever Hospital.

COTTAGE HOSPITAL, TETBURY.—Miss Florence Broome has been appointed Nurse. She has just completed a course of three years' training at St. Olave's Infirmary, Rotherhithe.

### Nursing in America: A Rejoinder.

BY AN AMERICAN GRADUATE.

(*Continued from page* 268.)

SOCIAL POSITION.

"AN ENGLISH NURSE" remarks that "she was not used to idleness." Neither are we in America. A good nurse there has more work than she can possibly do. "An English Nurse" must have a strange idea of the definition of the word "loyalty" if she thought she had only to go to America to pass over the heads of her fully-qualified American sisters and to supersede them with the medical men. Oh, no! that is not the *modus operandi* of the American practitioner. They prefer the nurses they know and whom they have, as it were, trained themselves, to foreign outsiders. Our life is not, as "An English Nurse" states, a lonely one. Nor can I let the statement made with regard to our social position there go without indignantly contradicting it. During my thirteen years of nursing in America I never had my meals offered to me elsewhere than with the family of the patient I was in charge of.

THE BUSINESS OF NURSING.

The happiest days of my life were spent in hospital, and many of my fellow-graduates—from choice, not necessity—remain there after receiving their diplomas. I cannot help feeling sorry for poor "English Nurse's" conceit. She could not realise that her American sisters were slightly sarcastic when they pitied her and enlarged on her "great experience." We don't take to nursing in the States simply as a means of making money; but even granted that we did do so, our reward is far in excess of the remuneration offered to "An English Nurse."

DAY DUTY—TEMPERATURE.

The hospital in the mountains must have been a most inferior one—more on the nursing home principle we find in England. The matrons of my experience would not have asked a nurse, even if she were a graduate, "if she minded coming on day duty." Lord Roberts might just as well ask one of his officers if he would mind going with his regiment into action. The matron is a nurse's head authority; the nurse is there to obey, not to object. Unless by special order, every nurse knows that the temperature of a ward is usually 65 deg. to 68 deg. Then why on earth did not "An English Nurse" ventilate her ward and so regulate her temperature?

TIRESOME TRAYS AND DIETS.

Of course, it is every nurse's duty to prepare special diets. If English nurses wonld only take a little more little more interest in these diets, and in the dainty preparation of trays, they would see a greater improvement in their patients, and find that their food was far more enjoyable when served up in an appetising manner.

HARD WORK.

The fact is that "An English Nurse" does not know the full meaning of hard work, and her nursing must have been of a very inferior kind if she omitted her medicines and temperatures. Such is not my record; and I never heard of a doctor giving orders to anyone but the "nurse in charge," unless he thought she was not competent to receive them. Neither would I dream of taking a doctor's orders second-hand from a patient. The nurse in charge making rounds with the doctors carries the ward book, and the senior resident doctor enters the orders in it as they are given at each bed; and every bed has its own chart with name, orders, and medicines and diets with rules written thereon. I had the pleasure of nursing for a leading London physician some time ago, and he asked me as a great favour for a copy of my notes, to teach, as he said, the English nurses how to keep them. No properly-equipped American hospital cooks the food of private patients in the main kitchen. It is all prepared in the diet kitchen; of course, each nurse has charge of her own trays. If "An English Nurse" never had trays before to prepare, this was an excellent opportunity for learning a very dainty piece of work. I never heard of any hospital nurse having their dinners after or off patients' joints, or cooked in the patients' kitchen. Such a thing is preposterous.

LINEN CLOSETS, BATH-ROOMS, &C.

It is a nurse's pride to keep these neat, and the linen closets are generally in the charge of the junior nurse. She is also responsible for their neatness while she is a junior, which is the first six months of her hospital life. Lavatories and baths in any American hospital where I have been were kept spotless. I have been ashamed and disgusted to see the condition in which some of the English hospitals keep their bath-rooms, &c. It has been a work of months to change the daily untidy, filthy state of things there into antiseptic cleanliness. Our patients were thoroughly washed each day, and the bathing of them was not, as "An English Nurse" describes it, "a movable feast." Each junior nurse and probationer in the wards has so many patients given to her each week to bathe, and for the cleanliness of these she is responsible to the head nurse. The senior nurse bathes the six sickest patients. The head nurse or sister-in-charge makes out the bath list every Monday, with the name of the patient and the description of bath the same is to get, whether tub, sponge, or bed bath. This list is hung up, and as each patient is bathed the name is crossed out. Every new patient gets a bath at once on entering hospital. There is no question of a nurse not having time to give the baths. She must have time, and no patient is allowed to refuse a bath. The American people are very fond indeed of bathing, and even in the poorest private houses the workman has his bath-room. Very different is it in England, where one rarely finds bathrooms, and where, even in the hospitals, patients continually tell you they "never had a bath in their life."

HOURS OFF DUTY.

Each nurse has in the hospital her own regular time off duty. If this has to be changed it is done by the sister in charge, and every nurse is compelled to spend some of her off-duty time each day in the open air unless she is ill.

INFLUENZA.

It is not a very laborious process to feed up an influenza patient, and surely half an hour of massage twice a day would not prostrate any nurse. I can only conclude it was a most fortunate thing for all concerned that "An English Nurse" did fall a victim to influenza, and so decided to leave the hospital and return to her English home. If "An English Nurse" could only obtain three dollars a week for her nursing the American people could not have thought much of her capabilities. I have never experienced the slightest difficulty in getting twenty-five dollars a week, and people never dreamed of expecting me to do their housework. They were perfectly aware I was a nurse, not a servant. At the same time, I do not consider any work a nurse may have to do, if it be for her patient's comfort, is in any way detrimental or lowering to herself or her profession as a nurse. In conclusion, I would strongly advise "An English Nurse" to remain in England, where she won't be ashamed of her sister nurses, and where she won't be so hard-worked. For myself, I would not change my American training for all the English certificates that could be offered me.

### Echoes from the Outside World.

### AN OPEN LETTER TO A HOSPITAL NURSE.

Intense relief has been afforded to the country, and still more to those who have relatives or friends in Peking, by the announcement that the Legations are safe. It is not surprising that many persons declined to accept the report until it was officially authenticated, for ever since the Chinese crisis arose, the news has been of an extremely conflicting character. Happily, the Queen has been able to address a characteristically sympathetic telegram to the officer commanding the Royal Marine Guard at Peking, expressing her thankfulness that he and those under his command are rescued from their perilous situation. Now that the story of the massacre of ministers is finally disproved, it is very proper that those journals, which, not content with chronicling what nearly everyone supposed to be a terrible fact, professed to possess exclusive information and entered into sensational details, should be called over the coals. You may remember the following "vivid account" in one of the dailies: "Thus standing together, as the sun rose fully, the little remaining band, all Europeans, met death stubbornly. There was a desperate hand-to-hand encounter. The Chinese lost heavily, but as one man fell others advanced, and finally, overcome by overwhelming odds, every one of the Europeans remaining was put to the sword in a most atrocious manner." This harrowing word picture was in the circumstances assumed by Sir John Scott and many other persons to have been a Fleet Street concoction, or, in more ambiguous language, the expansion of a telegram. But it appears to be beyond doubt that the story was transmitted by some one in the Far East, and was published in good faith. None the less is it true that it has exposed us to the charge of slandering the Chinese wholesale. As it is obviously not true that "every one of the Europeans remaining was put to the sword in the most atrocious manner," no apology to the nation which has been maligned can be too definite. Although no legal tribunal can be invoked in such a case as this, one cannot help thinking that the offence of libelling a single individual is far less heinous than that of traducing a people.

---

It is understood that Clarence House will be assigned by the Queen to the Duke and Duchess of York. The widowed Duchess of Saxe-Coburg is not likely to spend much of her time in England; and that part of St. James's Palace which is known as York House is, at best, only a makeshift residence for the Heir Presumptive to the Throne and his increasing family. Clarence House, on the other hand, is most suitable, and will afford ample accommodation. The only other member of the Royal Family who has been mentioned as the possible occupant of the mansion is the Duke of Connaught. But so long as he is in command at Dublin he will seldom be in London, and both the Duchess and himself are well content with beautiful Bagshot.

---

Possibly because they have more leisure to devote to accomplishments than those who are obliged to train themselves for business so as to make a livelihood, the Royal folks of Europe seem to shine in the artistic world. At the sale of pictures in the spring for the benefit of the War Fund (which, by the way, do you see, has now reached a million pounds), there were works by the Prince Consort painted many years before, and also by our Queen herself. The skill of the Princess Louise as a sculptor is well known, and Princess Beatrice is clever with her brush. At the Grand National Bazaar there were several artistic contributions by the German Emperor, and now I notice that King Carlos I. of Portugal has been a prizewinner in the Fine Art section of the Paris Exhibition. The jury were at first much exercised in their minds as to how they should treat their royal competitor—whether he should be looked upon as a king, in which case I suppose he would have been placed in the front rank quite irrespective of his merit, or whether he should be treated as an ordinary personage and judged entirely with regard to his artistic ability. France being a Republic, they naturally determined to adopt the latter course, and have therefore placed King Carlos in the second class, and given him a silver medal for his pastel. Doubtless he will value such an award won in fair field much more than the most gorgeous golden trophy presented to him owing to the accident of his being a monarch.

---

Whereas I generally write to you from an ordinary street in the metropolis, I am now sitting at an open window, overlooking the most beautiful panorama of green pastures, golden cornfields, waving woods, and unploughed land of a deep-coloured red earth, contrasting exquisitely with the other hues around. The air is sweet and fresh, with just the touch of ozone in it which makes one feel conscious of drinking in health at every breath, and the perfect stillness is only broken by the hum of the bees, the song of the birds, and the occasional lowing of distant cattle. How some of the weary toilers up and down the wards of the busy hospitals would love to find themselves in this blissful, lazy land, and how I wish I could send you with my letter a few of our refreshing breezes! We are in Devonshire, at a farmhouse between Sidmouth and Budleigh Salterton, and all around is so pleasant and so beautiful that it is a joy to live. I wonder if you know this part of the country? The coast is bounded by very high cliffs, not the pure white chalk which you find in so many places, but deep terra-cotta sand, overgrown here and there by gorse and fern and flowering plants and shrubs. The heights are tremendous, and the views correspondingly grand, whilst the sea is more changeful than usual. Sometimes it looks grey, and dull and dead; at others a brilliant blue; and then, in the next hour, a clear transparent green. The beach is almost deserted just where we go, it is so far into the country, but, of course, there are plenty of visitors at Budleigh Salterton and Sidmouth.

---

I must tell you about the holiday camp which has just broken up at a farmhouse near here. It consisted of twenty-five members of the Young Men's Christian Association, who had come down from London. They brought their own hammocks, which were slung four deep between the beams of a large barn, over 100 feet long, running all down one side of the farmyard. The floor was covered with clean, new sacks, which made a comfortable carpet, and plenty of hanging lamps lighted up the apartment at night. All day long the young fellows were out in the air, returning ravenous to the wholesome meals provided by the good wife at the farm, who, when the weather was fine, gave them their meals out of doors. Otherwise they were accommodated in the large kitchen, which, had I been an artist, I should have started at once to sketch. There is no range, only a large, square, open fireplace, the fire itself being fed with sticks, which blaze up and boil the large iron cauldrons hanging, gipsy-like, over the flames. The joints are frequently cooked in one of these pots, covered lightly with hot ashes, taken every few minutes from the fire and piled well over the simmering meat. By the fire is a polished wood settle with a high back to keep off the draught, and cushioned all along, big enough to hold eight or ten people.

### Private Nursing in Rhodesia.

### INTERVIEW WITH MRS. NELL.

By a Correspondent.

I have just been hearing about a private nurse's work in and near Bulawayo from Mrs. Nell, who came to England in charge of the "Avondale Castle." She is convinced that there are excellent openings for good private nurses in Rhodesia, where the doctors find it difficult to get nurses they can trust. But they must be thoroughly trained in all branches of their profession.

### What the Cases are Like.

"Most of the cases," she said, "are midwifery ones. The country is very healthy, and unless people happen to live in low or marshy districts, where fever is easily contracted, they do not often suffer from illness. The air is not full of bacilli, as it is in London, for instance. I was trained at Westminster Hospital, but I obtained my L.O.S. certificate before going out."

"And then did you join a nursing home?" I enquired.

"No. I did not want to bind myself, and as it happened that I was nursing in Cape Town, with a lady who lived in Bulawayo, I went with her there, and then started on my own account."

"Did you find the plan successful?"

"Very; I was never without a patient from that time until my marriage. I simply went from one to another."

"I suppose," I suggested, "that if a nurse was out of work often living would be rather expensive?"

### Hard Work.

"I believe it would. I knew of a nurse who had a room kept for her at the hotel, and I fancy that would take a good deal from the profits. I can only say that it paid me well, for the fees are good—£5 5s. a week, and board and lodging. But the work is hard. For one thing, a patient very rarely has two nurses. Maternity cases, of course, mean day and night for the nurse."

"And the living may be rough sometimes?"

"Yes, the nurse has sometimes to do all the housework as well, though some English families have English servants with them. I had more than one lady to nurse where there were only little black piccanninies to help, and they were more trouble than they were worth."

"Had you often to travel great distances?"

"I had one case 110 miles away. It was an awful experience; the journey had to be made in a mule waggon, over rough country that English people have no conception of. But wherever it was practicable the doctors recommend their patients to come into the towns to be treated."

"That must be very trying to the patient if the case is sudden or serious?"

"It is; the hardships of the journey are terrible, especially if they do not come in early enough stage of the illness. But it is very much better to come if possible, for, of course, nothing can be got in a hurry out in the country. They usually take rooms in the hotel or a boarding-house, and have their own doctor and nurse. I once hired two tin rooms for a patient; they cost £8 a month. I had everything to do, even the cooking, and I had only two little black boys to hew wood and draw water."

### The Need of Training.

"Maternity cases want a thorough all-round training, because malaria very often comes out during confinement. This I did not know, until it came within my own experience. My patient was going on all right until the third or fourth day, and then she began to shiver, and I was in an agony for fear lest she had taken cold, though she had had every care. Well, I did all I could with hot bottles and blankets to keep her warm, and sent at once for the doctor. The first thing he asked her was, 'Have you ever had malaria?' 'Yes,' she replied, 'I had camp fever at Johannesburg.' So that explained it, and I found that it does sometimes occur at these times, when the patient has once had it. Some of the doctors get so accustomed to prescribing for fever that they are apt to forget there are other ills. I knew an elderly gentleman, a manager of mines, who was on his way through the country inspecting the mines, and he stopped to get food at a wayside store. There was nothing to be had but pork almost raw, and a hard kind of sausage that might be anything. After his meal he felt very bad, and you can imagine how furious he was when the doctor treated him for fever!"

"There are hospitals at Bulawayo, Gwelo, and other places," continued Mrs. Nell. "The Bulawayo Hospital was formerly managed by nuns, but it did not answer very well, as they were not trained nurses, though they were very sweet and kind, and the doctors agitated for trained nurses: and now it is under a thoroughly organised system. I believe the nuns felt a difficulty about nursing men patients, and as the greater number are men, who contract fever while they are out on the veldt, it was well to make the change."

Mrs. Nell thinks that there is a very good opening in a new town like Bulawayo (the "old days," of which she referred once or twice, are only as far back as 1895) for a specially trained children's nurse, as most of the residents are young married people with families.

### The Black "Boys."

She herself has given up nursing since her marriage, and is devoting herself to a farm of five acres, on which she employs native labour. The photographs of the pretty bungalow, with its covered way between house and kitchen, and Mrs. Nell herself, wearing a large white "cappee" made for her by a Dutch woman, were most charming.

"I devote myself to doctoring the natives now," she said, laughingly. "They place the greatest faith in my remedies and bring all their babies and husband when anything is the matter, and as they are terribly frightened if they have the least thing the matter with them, I get plenty of practice. They are great cowards, and if a boy cuts his finger he will lie all day in the hut thinking he is going to die. Their fear of death is intense. Happily, very simple medicines usually put them right, and the nastier the 'mooti' is the better they like it."

### The Voyage Home.

Finally, Mrs. Nell told me that she had by no means intended coming to the old country when she left her home in Buluwayo; she was becoming seriously ill through the food, which was both dear and bad, owing to the war, and she went to Cape Town for a change, where the railway was reopened through ruined Mafeking. "Cape Town, however," Mrs. Nell said, "never suits me well, and as I got the offer of the voyage in charge of the sick and wounded on board the 'Avondale Castle,' I accepted it, and came home. We had a very pleasant but uneventful voyage."

"You are returning, though?" I inquired.

"Oh yes, probably at the end of the summer. Six months' leave is granted by the War Office. The climate out there is so splendid that I long to go back to it, though it is delightful to pay a visit to the old country."

### Death in Our Ranks.

We regret to record the death of Sister Dora, formerly night superintendent at the Manchester Royal Infirmary, sister at the Royal Infirmary, Leeds, and nursing sister to All Saints' Parish, Scarborough. Sister Dora had been an invalid for the last five or six years.

### A Journey from Capetown to Pretoria.

### FROM WYNBERG TO PRETORIA.

By a Nursing Sister.

After spending some time at Wynberg we left Capetown, as we then thought, for Bloemfontein. At eight a.m. on Sunday we got out at Magersfontein, had breakfast, and then visited the officers' convalescent home, an exceedingly comfortable place; it is a hotel lent or hired for the purpose, and superintended by a medical officer and his wife, who was formerly a hospital sister. Magersfontein was, I think, the neatest little village we passed. On resuming our journey we contented ourselves with viewing the country, and very dreary it looked, nothing but veldt and kopjes, with here and there a few mud huts of Kaffirs to-day in their Sunday best. The names of many of the places we passed were very familiar from newspaper fame. A heavy rain began to fall in the afternoon, and one realised how terrible the marches must have been for our poor men on this rough, stony veldt. At eight p.m. we arrived at Victoria West, where we were to dine. The dining hall is a little way out of the station. So, well enveloped in mackintoshes, we started, running into a barbed wire fence before we arrived. The food is horrible at these places, and we had the greatest difficulty in getting clean plates, &c. On returning to the train we found the rain coming into our carriage from the roof, and had to place sponges and towels to soak it up. We had previously heard that a train going up a few days before had been "sniped" by the Boers; we were told that our lights would be put out instantly if an alarm was raised. About four o'clock my companion awoke me, saying the lights were out. She suggested we should go under the seat if firing began! I was much too comfortable to move, and I think we both fell asleep in a very short space of time. At eight a.m. we breakfasted at Norvals Pont, and I had the pleasure of seeing my Edinburgh friends. The country looked prettier here. At Springfontein I saw one of the Welsh sisters; they have had a sad time, losing so many of their staff. The rain still continued; the country was getting much flatter. There were several scouts about, and numbers of dead horses lying about, telling their own tale. How one did pity the poor sentries who were guarding the lines, with everything drenched. We were now nearing Bloemfontein. The outlook was not inviting, and we dismally wondered where we should go, having received no definite instructions with regard to our destination, and it was too late to see the P.M.O.

### Arrival at Bloemfontein.

By the time we had arrived at Bloemfontein the platform was crowded with baggage, khaki, and Kaffirs. We consulted the staff officer, and great was our joy when we were told to go on to Pretoria in Lady Roberts' special train. We had just time to get some dinner and then entrained again. Several more half-drowned sisters joined us, and I believe Lady Roberts and her two daughters boarded the train that night. But we were not allowed to travel at night owing to the line having been so recently cut. At six a.m. on Tuesday we left Bloemfontein with an armoured train in front of us. We were on rations, and some comical meals we had. We had to be sparing, not knowing how long we might be on the way. And our journey began to be exciting. Every bridge we came to had been blown up; we crossed over temporary ones, which simply made one gasp till you were safely over.

### Lady Roberts Dispenses Tobacco.

We were constantly passing camps, and I think Lady Roberts made every man happy by throwing out tobacco, &c. The country was still very flat, and such lots of dead horses lying about. Virginia siding, by the Zand River, is very pretty. Numbers of "Tommies" were busy repairing the lines and rebuilding the bridge. We were taken down a steep cutting, owing to the line having been cut; every now and again we got a glimpse of our armoured train. In the afternoon we arrived at Kroonstadt, where we were to stay the night.

### A Visit to No. 3 General Hospital.

I visited No. 3 General Hospital, having some friends there among the sisters. It was very wet, and I found them working in macintoshes and goloshes. Their marquees were all very full, and they were very short of water and not too well off for provisions. Most of the sisters like the camp life excepting in wet weather, bedclothes and everything getting damp. Then the mud! Shall I ever forget the mud of South Africa? I had tea with the sisters and bread and jam. Butter is an almost unknown luxury; at Pretoria it is 5s. 6d. per pound.

### The Scottish Red Cross Hospital.

Afterwards I visited the Scottish Red Cross Hospital, and found my friend busily preparing a bath in one of her marquees. Ninety patients had just arrived, some of them wounded, but I was not able to ascertain where they came from. This hospital is splendidly equipped. We started off again early in the morning, and saw the place where the Railway Pioneers were recently attacked, also the graves of the killed. I secured a piece of shell here. Later on we arrived at Roodeval, where the mail bags were taken and the poor Derbys cut up. There were a lot of men waiting to be picked up by a train going down country. They had burned De Wett's farm previously. They were very badly off, having lost a good many of their things. We gave them what provisions we could, also scraps of writing paper, soap, &c. Lady Roberts showered tobacco on them to their great delight.

### British Prisoners in Boer Garments.

At one of the stations we saw and spoke to some of the men who were captured at Stormberg; they were dressed in various articles of Boer clothing, and some of them were very much emaciated, their rations while imprisoned (for six months) being chiefly mealie food, with one pound of meat once a week and tea once a month! We also saw some Boer prisoners brought in at one of the stations. The stations are all occupied by military people, waiting-rooms are turned into offices, &c. About the middle of the day we were kept waiting for a long time; afterwards we heard that Boers had been on the line, but nothing exciting happened. Subsequently crossing the Vaal the country became prettier. Some sisters who were with us began to prepare to leave us at Elaandsfontein. They were going on to Johannesberg; Lady Roberts got out to wish them God-speed. We were rather disappointed that we did not go round to Johannesberg.

### Arrival at Pretoria.

About seven p.m. we steamed into Pretoria. Lord Roberts, Lord Kitchener, and staff were at the station to meet Lady Roberts. After the family greeting was over Lord Roberts came and shook hands with us, and told us we were greatly needed in Pretoria. What a proud moment it was! How delightful it was to get such a gracious welcome from the Commander-in-Chief. Next morning we were sent to the Palace of Justice Hospital. The Irish Hospital is in charge just now, the building is the new Law Courts, which Mr. Kruger was to have opened in January. It is a handsome structure, and accommodates between five and six hundred patients. On Wednesday, July 11th, Lord Roberts opened the building for its present purpose. While he was making his speech distinct firing was heard; later we heard of the disaster to the Lincolns and Scots Greys, only six miles from here. From the tower we can see the Boer positions. A great many troops have since left, also Lord Roberts. It is very sad to think it is not all over yet. Of Pretoria I have not seen much, being very busy. The houses have lovely gardens, and the surroundings are also very pretty. There are a great number of Tommies about—Tommies who would never be recognised at home, so scorched and ragged and dirty they are. Our soldiers will never, never forget South Africa and its hardships.

### Everybody's Opinion.

[Correspondence on all subjects is invited, but we cannot in any way be responsible for the opinions expressed by our correspondents. No communication can be entertained if the name and address of the correspondent is not given, as a guarantee of good faith but not necessarily for publication, or unless one side of the paper only is written on.]

### A USEFUL HINT TO NURSES.

"J. R." writes: Have any of you come off duty at night feeling very tired and footsore after an extra busy day in the wards? If so and you care to try the following recipe you will find it a splendid thing for tired feet, and one that is not generally known. Soak your feet in tepid water in which has been dissolved a good handful of salt for twenty minutes or half an hour; do not dry them, but let the moisture soak well in. If you do this for a few days in succession you will find it will harden your feet wonderfully. I have tried it, and can, therefore, speak from experience.

### A LABOUR OF LOVE.

NURSE BEATRICE writes: There is one branch of our profession which I am afraid we are apt to shrink from as being "so monotonous and uninteresting." I refer to the nursing of chronic cases, of which there are alas! so many. They always strike me as being so sad. The once active body laid low and helpless, the horizon of life narrowed, is it to be wondered at if the patients do sometimes get soured? Should we not rather think it a privilege if we can bring a ray of comfort, however small, into these darkened lives? We need not think our skill wasted if we can in a little measure alleviate their sufferings. I think I have given a large portion of my sympathy to these poor hopeless cases. Surely they have as much need of trained care as an "enteric." The path which we have chosen entails a vast amount of self-sacrifice, and if we are to be of any real help to the sick and suffering what right have we to consider our own feelings first? Of course, we naturally like the excitement of a "good case," and a hand to hand fight with death. But there is not room for us all "at the front," much as we would like to be there. Shall we not try and shine just as brightly in our little corner as the devoted sisters who are braving everything for the sake of "Tommy"?

### NURSING IN AMERICA.

"AN ADVOCATE OF TOLERANCE" writes: I feel that I ought to protest against the assertion made by "An American Graduate" that "the English doctor seems to forget that a trained nurse is, or ought to be, a lady," and "treats her in most cases with less respect than he would show his housemaid." As an Irishwoman I know that our national fault is a too generous enthusiasm, which carries us away in our desire to show loyalty where it is justly owed. But I could not allow such a stricture as this to pass without giving my experience to the contrary; it may also be that of the newer generation. After my training I worked on for a year as private staff nurse to my hospital before leaving, and in that time met with unfailing kindness and consideration from every individual doctor under whom I nursed. I shall always remember with pleasure the kindly interest and courtesy of one of our best-known northern specialists, whom it was my privilege to meet at a case, and who has since laid down his life in service to the sick and wounded in South Africa. As to the statement that our American sisters are far superior to us in their nursing capacity, it would be doing us justice on the part of "An American Graduate" to state which of our famous London schools she has tried and found inferior to St. Luke's?

### THE GENERAL HOSPITAL, MADRAS.—A GOVERNMENT INQUIRY ASKED FOR.

"ONE WHO HAS SUFFERED" writes: In the "Nursing Mirror" for July 28th "A Resident in Madras" called attention to the unsatisfactory state of affairs in the General Hospital, Madras, and stated that the real difficulty with regard to the nurses is the "keeping them." In last week's issue "A Member of the Committee of a large Provincial Hospital" says "if the official records of the General Hospital were examined it would be found that during the last two or three years a quite abnormal percentage of the English nurses and probationers have resigned prematurely." The nurses are then blamed for lack of moral courage. It is said that they do not state their real reasons for leaving. The writer is, I think, a little severe. It is never an easy, nor a pleasant thing to make an official complaint. In the General Hospital, Madras, it is especially difficult. I know of at least two nurses and one probationer who during the last two years have left the hospital, and have stated their reasons officially, with the result that in each case their characters have been attacked and their reputations have suffered. What can a working woman do then? The answer is, "Clear herself, of course." This is easier said than done. The General Hospital is a Government institution; it has no local committee. If a nurse's character is attacked she can appeal to the senior medical officer; if that fails, to the surgeon-general. If no redress is obtained there, further appeals may be made to the Government of Madras, to the Governor of Madras, the Government of India, the Viceroy, the Secretary of State for India, and I suppose, finally, to the Queen-Empress as supreme authority. But all this takes time and money. Meanwhile, how is the victim of slander to live? It is not easy for a woman with a good character to get work in these days of severe competition. What must be the fate of those whose reputations are smirched? If Sir Henry Burdett does take up the question and succeed in getting an unbiased inquiry held, he will win the gratitude of many helpless and shamefully-treated women.

### Appointments.

BELPER ISOLATION HOSPITAL.—Miss Louisa Freeman has been appointed Superintendent Nurse at this hospital. She was trained at the Shrewsbury Infirmary and the Chester Infirmary Isolation Hospital. She was staff nurse at King's College Hospital, and held appointments at the Sarah Acland Home, Oxford, and nursed during the Worthing epidemic. Most recently she was matron of the Borough Infectious Hospital, Kidderminster.

FOLESHILL INFECTIOUS HOSPITAL, NEAR COVENTRY.—Miss A. Newbold has been appointed Nurse-Matron of the above hospital. She was trained at the Isolation Hospital, Bagthorpe, Nottingham, and at the District Hospital, Batley, Yorks. She subsequently held appointments as charge nurse at the Milton Infectious Hospital, Portsmouth; the Borough Hospital, Croydon; and the Basford Sanatorium, Nottingham.

COTTAGE HOSPITAL, TETBURY.—Miss Amy Jones has been appointed Matron. She was trained at Fusehill Infirmary, Carlisle, and for three years at St. Olave's Infirmary, Rotherhithe. She has also been charge nurse at Newcastle-on-Tyne, Keighley, and Spalding Union Infirmaries for two years, one year, and eighteen months respectively.

PONTYPRIDD UNION INFIRMARY.—Miss Christina Fulton has been appointed Superintendent Nurse at this infirmary. She was trained at the Chester Infirmary, and was subsequently charge nurse at the Birkenhead Union Infirmary, sister at the Bootle Corporation Hospital, and district nurse at Nailsworth, Gloucestershire.

WELLINGBOROUGH HOSPITAL, HANTS.—Miss H. P. Berry has been appointed Matron of the above institution. She was trained at Guy's Hospital, and has since occupied the post of matron at St. Monica's Hospital, Easingwold, Yorkshire.

ROYAL INFIRMARY, SHEFFIELD.—Miss Edith Mary Shotier has been appointed Matron to this hospital. She has been occupied at the Sussex County Hospital, Brighton, as ward sister, theatre sister, and assistant matron successively.

SCARBOROUGH INFECTIOUS DISEASES HOSPITAL.—Miss Mary Pavyer has been appointed Matron. She was trained at the City Hospital East, Old Swan, Liverpool.

HORTON INFIRMARY, BANBURY.—Miss Wetherell has been appointed Matron. She was trained at the Nightingale Home, St. Thomas's Hospital.

### A Book and its Story.

### AN AMUSING NOVEL.

MANY reviews in praise of Mr. Harland's latest book* have already appeared, and we would add one more appreciation of this witty satire—a comedy of two—in which there is no dull page from start to finish. The hero tells his own story, and we smile with him, as he smiles at himself in the, at times, perverse absurdity of the situations and conversations. Never once, whether in homely, meditative moments, or in scenes threateningly near tragedy, which the alert reader perceives will end in comedy after all, does Peter Marchdale, with his persistent personality, disappear. He is found, in the opening chapter, at a rustic table sipping his steaming coffee, cigarette in hand, gazing at a scene which, as an author and artist, appeals instinctively to him. In the foreground, at his feet, lies a valley of loveliness, watered by the river Aco, to whose banks from the opposite side slopes down the smooth lawns of the park of Ventrois crowned by the turrets of the castle, in which the presiding genius of the district, his landlady, the young widowed Duchessa de Santiangiola, resides. To the right are dark forest-covered heights, and away in the distance at the valley's end gleam the snow-crowned summits of Monte Sfiorito, reflecting every passing effect of the day's varying mood.

"'It is not altogether a bad sort of view, is it?' said some-one, in English.

"The voice was a woman's. It was clear and smooth; it was crisp, but distinguished.

"*Vis-a-vis* to Peter, six or seven yards away, the river dividing them, stood the Duchessa, smiling.

"Peter's eyes met hers, took in her face," and in that moment's glance he knew that here, before him, with a little silver streak only between them, stood the lady of his dreams. The woman who had inspired him to idealise her as heroine of the book which had made his fame, who, seen once, was loved for ever. In fleeting vision she had crossed his path more than once—in Paris at the opera, later once or twice in London, then again in the Bois, and now, here. Until to-day he had never heard her voice, but in all his wanderings, her face, with its vague, dreamy spirituality, its unusual combination of sweetness and strength, its aliveness and refinement, its distinction, its humour, its capriciousness, generosity, emotion; all that indescribable *something* which marks personal individuality, was present.

In the past Pauline, as he had named her, had been a thing unknown, divined merely. Her nationality was uncertain even, although he imagined her an Englishwoman married to a foreigner. But here, by the direction of fate, he was brought face to face with his heroine, into her immediate vicinity.

From the sublime to the everyday is a descent that occurs naturally enough in a novel like the present. Peter returns after their brief interview to his garden seat his cigarette, and his reflections, the nature of which is suggested by the following extract of a conversation with his Italian housekeeper. "Old Marietta, the bravest of small figures, in her neat black and white peasant dress, with her silver ornaments and her red silk coif and apron, came for the coffee things. At the sight of Peter she abruptly halted. She struck an attitude of alarm. She fixed him with her fiery little black eyes."

"The signorina is not well!" she cried, in tones of one launching a denunciation.

Peter roused himself.

"Er—yes—I'm pretty well, thank you. I'm only dying," he added sweetly, after an instant's hesitation.

"Dying!" echoed Marietta, wild, aghast.

"Ah, but you can save my life. You come in the very nick of time," he said. "I'm dying of curiosity—dying to know something that you can tell me." . . . "Ah! the signorina gave me a fine fright," she said.

"A thousand regrets," said Peter. "Now, be a succouring angel, and make a clean breast of it. Who is my landlady?"

The required information being given, Peter quotes, in reply to Marrietta's confusion, a phrase he had read in a recent novel, "I rather think she is my long-lost brother."

"Brother?" faltered Marietta.

"Well, certainly not sister," said Peter, with determination. "You have my permission to take away the coffee things."

"In her rose and white boudoir at the castle the Duchessa was writing a letter to a friend in England. 'Villa Gloriand,' she wrote, 'is let to an Englishman—a youngish, presentable-looking creature in a dinner jacket, with a tongue in his head, an indulgent eye for nature, named Peter Marchdale.'"

From this it is inferred that the adored one was not struck at first sight with Peter's adorable qualities, which lay not in any surface attractiveness especially, but rather in intellectual gifts, to which, on further acquaintance, the Duchessa was by no means indifferent. United to a certain tough manliness and an absurd sensitiveness typical of the ordinary Englishman, not in themselves drawbacks in a lover whose lady has humour and perception sufficient to read between the lines, as the Duchessa had. The comedy runs on, with diffidence on Peter's part—his position being one of increasing difficulty. The Duchessa realises also that there are difficulties. His fear arises from the barriers which wealth, and position place between them; hers from the difference of Faith alone. She has an uncle, the Cardinal Udeschina, a wise and witty ecclesiastic; he must be consulted.

"The priest sat in an arm-chair, smiling amusement, indulgence; the Duchessa, critical-eyed, stood before a pier-glass between two tall windows, turning her head from side to side, examining (if I must confess it) the effect of her new hat. 'Well? what do you think?' 'Well, then, I think if the feather . . .' 'Good gracious, I don't mean my hat,' said Beatrice. 'What in the world can an old dear like you know about hats? I want to know what you think not of my hat, but of my man?' 'Oh, ah, yes; your Englishman, your tenant. . . .' 'Ah, please don't tease about this; I want to know what you think of his conversion?' 'The conversion of a heretic is always a consummation devoutly to be desired.'" The Cardinal returns with his niece to Ventiroso, and on an incident connected with his snuff-box, the consummation of the story, as well as the title of the book turns. A clever, entertaining, and very readable romance.

* "The Cardinal's Snuff-Box." By Henry Harland. (Publisher, John Lane, London and New York. One Vol. 6s.)

### To Nurses.

WE invite contributions from any of our readers, and shall be glad to pay for "Notes on News from the Nursing World," or for articles describing nursing experiences, or dealing with any nursing question from an original point of view. The minimum payment for contributions is 5s., but we welcome interesting contributions of a column, or a page, in length. It may be added that notices of entertainments, presentations, and deaths are not paid for, but, of course, we are always glad to receive them. All rejected manuscripts are returned in due course, and all payments for manuscripts used are made as early as possible at the beginning of each quarter.

### For Reading to the Sick.

PITY makes the world soft to the weak and noble to the strong.—*E. Arnold.*

The truest joys which we have experienced have come when we have had grace to enter most entirely into a sorrow not our own.—*Westcott.*

No stream from its source
Flows seaward, how lonely soever its course,
But what some land is gladden'd! No star ever rose
And set, without influence somewhere! Who knows
What earth needs from earth's lowest creature? No life
Can be pure in its purpose and strong in its strife,
And all life not be purer and stronger thereby!
The spirits of just men made perfect on high—
The army of martyrs who stand by the Throne
And gaze into the Face that makes glorious their own—
Know this, surely, at last! Honest love, honest sorrow,
Honest work for the day, honest hope for the morrow,
Are these worth nothing more than the hand they make weary—
The heart they have sadden'd—the life they leave dreary?
Hush! The sevenfold heavens to the voice of the Spirit
Echo: "He that o'ercometh shall all things inherit."
—*Lytton.*

**Reading.**

We look down when we ought to be looking up, we lose heart when we ought to be gathering new courage. "I could have borne up," cries the soul on the brink of despair, "if only I had not been so thoroughly alone, so utterly forsaken. I could have borne up, I could have been brave and strong, if only there had been one heart into which I could have poured my heart."

How many a life has become blank, pitiless, wasted, drifting down and down with the tide, just because when clouds gathered and life was hard, and its path so rough and edged with thorns, it was felt "I had no place to flee unto, and no man cared for my soul."

But, remember, it is because of that death, and because of the experience of these seven words which He cried from His cross, that there is one thing which you and I can always claim, and which claiming we are sure of receiving, and that is the perfect sympathy of the living Christ. Ought it not to be a real strength to us? Who can despair, who can fail in the end, who can be beaten, who has the inexhaustible store of human sympathy in the heart of God, into which he can ever cast himself? It is, indeed, a great and a strong reality, this perfect sympathy of Jesus in every human life, because of the cross, because of the experience of the seven words.

And if for any one of us the day should come when active work is no longer possible—if we are laid aside in broken health, if we are permitted to live on until age becomes feeble—well, let us remember then that "they also serve, who only stand and wait," and that "who best bear His mild yoke, they serve Him best."

Whether life for us be long or short, oh! the joy of bringing back our work to Him Who gave it to us to do, done, however imperfectly, in the strength of His great sympathy! —*S. W. Isaacs.*

Those learn what faith and love can do
When they to God and man are true,
And how that God approves
The deep devotion which would share
The burden of another's care,
And ever tru[illegible]s and loves.—*S. B. Monsell.*

### Notes and Queries.

**THE Editor is always willing to answer in this column, without any fee, all reasonable questions, as soon as possible.**

**But the following rules must be carefully observed:—**

**1. Every communication must be accompanied by the name and address of the writer.**

**2. The question must always bear upon nursing, directly or indirectly.**

**If an answer is required by letter a fee of half-a-crown must be enclosed with the note containing the inquiry.**

*Dispenser.*

(215) Will you kindly tell me how to secure a post as dispenser to one of the hospitals or nursing stations in South Africa? I am sole dispenser at this infirmary. I have also been trained as nurse, but am not fully qualified.—*Apothecary.*

You might apply to the Central British Red Cross Committee, 18, Victoria Street, Westminster, and to any of the hospitals sent out by private benevolence.

*Dispensing.*

(216) A lady, holding the Apothecaries' Hall certificate, would be very glad to know how to obtain a situation. She would go abroad if necessary. Can you help her?—*Christina P.*

She can only advertise, and watch the advertisements in the medical and municipal journals.

*Insane.*

(217) 1. Can you tell me of an asylum in Suffolk or elsewhere where for a small weekly payment I can place a woman who is becoming insane? 2. Is it necessary to have more than her medical attendant to certify that she is insane?—*E. A.*

It is possible that the After-Care Association for Persons Discharged from Asylums, Church House, Dean's Yard, Westminster, could recommend a private home for the case if it is not more desirable to place her at once with the local guardians. 2. Two doctors must sign the certificate if the patient goes to a private asylum; but if the guardians undertake the case their officials will arrange about the certificate.

*L.O.S.*

(218) Will you kindly advise me as to the best book to study for the L.O.S. examination?—*Would-be District Nurse.*

"A Practical Handbook of Midwifery," by Haultain, is one of the most highly esteemed. (Scientific Press, 6s.)

*Mental Nurse.*

(219) Will you kindly tell me if there is any home where they take certificated mental nurses without hospital training?—*H. B.*

We do not know of any home where a mental nurse's certificate would be accepted in place of one of general training.

*Trained in India.*

(220) Would a three years' certificate from a general hospital of over 100 beds in India enable the holder to get a good post in an English hospital, or would an English training be necessary?—*Sister C.*

You will probably be able to get a post in one of the large poor law infirmaries; but whether you would be allowed with such a certificate to rise to the higher posts can only be decided by the Local Government Board. See our advertisements.

*Supplementary Training.*

(221) Would you kindly tell me if there is any hospital in or near London where I could attend lectures or got postal preparation for certificate? I have had three years' experience in an infirmary asylum, and have taken the L.O.S. certificate. I have now charge of a small home for old people, and should be much obliged for any help towards obtaining a certificate for general nursing.—*Ivy.*

You cannot obtain a reputable certificate for general nursing except from a hospital where you have actually been trained.

*Specialists.*

(222) Would you kindly tell me who are the best London specialists for nervous disorders, and what their addresses and hours of consultation are? If this question is unsuited to your columns will you tell me how to find out?—*Queen's Nurse.*

You may easily obtain the information you desire by consulting the lists of physicians attending the large London hospitals.

*Books for Mothers.*

(223) What is the best book for advice to young mothers?—*E. M.*

Chevasse's "Advice to Mothers" has an excellent reputation. The Scientific Press, London, W., would procure it for you. Price 2s. 6d. This firm publishes at 2s. a nice little volume, entitled "The Mother's Help and Guide to the Domestic Management of her Children."

*Almshouses.*

(224) Will you kindly tell me where I can get all information abou almshouses?—*Miss A.*

"Burdett's Hospitals and Charities" for 1900 is the most complete list published. You may get it of tne Scientific Press, W.C., price 5s., if it is not in the public library.

**Standard Books of Reference.**

**"The Nursing Profession: How and Where to Train." 2s. net.; post free, 2s. 4d.**

**"The Nurses' Dictionary of Medical Terms." 2s.**
**"Burdett's Series of Nursing Text-Books." 1s. each.**
**"A Handbook for Nurses." (Illustrated.) 5s.**
**"Nursing: Its Theory and Practice." New Edition. 3s. 6d.**
**"Helps in Sickness and to Health." Fifteenth Thousand. 5s.**
**"The Physiological Feeding of Infants." 1s.**
**"The Physiological Nursery Chart." 1s.**

**All these are published by THE SCIENTIFIC PRESS, Ltd., and may be obtained through any bookseller or direct from the publishers, 28 & 29, Southampton Street, London, W.C.**

